# Establishment of a lethal mouse model of emerging tick-borne orthonairovirus infections

**DOI:** 10.1371/journal.ppat.1012101

**Published:** 2024-03-19

**Authors:** Takuma Ariizumi, Koshiro Tabata, Yukari Itakura, Hiroko Kobayashi, William W. Hall, Michihito Sasaki, Hirofumi Sawa, Keita Matsuno, Yasuko Orba

**Affiliations:** 1 Division of Molecular Pathobiology, International Institute for Zoonosis Control, Hokkaido University, Sapporo, Japan; 2 Institute for Vaccine Research and Development, Hokkaido University, Sapporo, Japan; 3 National Virus Reference Laboratory, University College Dublin, Belfield, Dublin, 4, Ireland; 4 Global Virus Network, Baltimore, Maryland, United States of America; 5 International Collaboration Unit, International Institute for Zoonosis Control, Hokkaido University, Sapporo, Japan; 6 One Health Research Center, Hokkaido University, Sapporo, Japan; 7 Division of Risk Analysis and Management, International Institute for Zoonosis Control, Hokkaido University, Sapporo, Japan; University of Pennsylvania, UNITED STATES

## Abstract

Emerging and reemerging tick-borne virus infections caused by orthonairoviruses (family *Nairoviridae*), which are genetically distinct from Crimean-Congo hemorrhagic fever virus, have been recently reported in East Asia. Here, we have established a mouse infection model using type-I/II interferon receptor-knockout mice (AG129 mice) both for a better understanding of the pathogenesis of these infections and validation of antiviral agents using Yezo virus (YEZV), a novel orthonairovirus causing febrile illnesses associated with tick bites in Japan and China. YEZV-inoculated AG129 mice developed hepatitis with body weight loss and died by 6 days post infection. Blood biochemistry tests showed elevated liver enzyme levels, similar to YEZV-infected human patients. AG129 mice treated with favipiravir survived lethal YEZV infection, demonstrating the anti-YEZV effect of this drug. The present mouse model will help us better understand the pathogenicity of the emerging tick-borne orthonairoviruses and the development of specific antiviral agents for their treatment.

## Author summary

Establishment of animal models are pivotal in the study of newly emerging infectious diseases. In this report, we show that mice lacking interferon responses can serve as a lethal animal model for emerging tick-borne orthonairovirus infections. Yezo virus is a tick-borne orthonairovirus that is genetically distinct from previously reported human pathogenic orthonairoviruses such as Crimean-Congo Hemorrhagic fever virus, and has recently been reported to cause acute febrile illnesses in humans in Japan and China. Type-I/II interferon receptor-knockout mice (AG129 mice) infected with Yezo virus developed acute disease with elevated liver enzyme levels, including AST and ALT, similar to human patients. Our pathological and virological analyses in this mouse model revealed that Yezo virus mainly replicates in the liver and spleen and causes fatal hepatitis in AG129 mice. Furthermore, we could demonstrate the antiviral effect of favipiravir for Yezo virus infection. Thus, this animal model will facilitate studies of emerging tick-borne orthonairovirus infections which will allow a better understanding of disease pathogenesis and will permit the screening of potential antiviral agents.

## Introduction

The genus *Orthonairovirus* in the family *Nairoviridae* consists of 52 virus species [1]. Certain orthonairoviruses are well known as human and/or animal pathogens. These include Crimean-Congo hemorrhagic fever virus (CCHFV) [2], Nairobi sheep disease virus [3], Dugbe virus [4], Kasokero virus [5], Issyk-Kul virus [6], and Erve virus [7]. Recently, emerging and reemerging human infections of tick-borne orthonairoviruses belonging to Tamdy or Sulina genogroups, which are phylogenetically distinct from previously reported human pathogenic orthonairoviruses have been identified. These include Tamdy virus [8], Tǎchéng tick virus-1 [9,10], Songling virus [11], and Yezo virus (YEZV) [12,13] and have been reported in China and Japan. These emerging and reemerging tick-borne orthonairoviruses have also highlighted the potential public health importance associated with the Tamdy and Sulina genogroups.

YEZV, an emerging orthonairovirus in the Sulina group was originally isolated from febrile patients who had a history of tick bites in Hokkaido, Japan. To date, eight infected patients have been reported, including retrospectively identified patients in both Japan and China [12,13]. In two clinical cases, acute fever with thrombocytopenia, leukopenia, and elevation of liver enzymes, creatine kinase, and ferritin levels were reported. But, no pathological studies have been conducted in human patients infected with YEZV or those with the other emerging orthonairoviruses including Tamdy virus, Tǎchéng tick virus-1, and Songling virus.

Previously reported small animal infection models of orthonairoviruses, such as CCHFV have been established using immunodeficient mice, such as interferon receptor knock-out mice [14–21]. In these models, the mice exhibit a variety of pathogenic features including liver damage, gastrointestinal disorders, and neurovirulence. Notably liver damage is a representative pathological feature commonly found in a variety of orthonairovirus-infected mice [15–18,20–22] and also in human CCHF patients [2,23–26]. Elevated levels of liver enzymes in YEZV patients suggest that YEZV infection may also potentially cause liver damage in humans. To investigate the pathology of YEZV infection, we have attempted to establish an animal model for YEZV infection. Specifically, we have developed a lethal mouse model of YEZV infection using type-I/II interferon receptor-knockout mice (AG129 mice) [27] for pathological analysis and for validation of potential antiviral agents. Histopathological and virological analysis using this model revealed that YEZV replicates primarily in liver and spleen resulting in a fatal hepatitis. Furthermore, AG129 mice treated with favipiravir (T-705) were rescued from lethal YEZV infection thereby successfully demonstrating the antiviral effect of T-705 in this mouse model. The present study is the first small animal model for YEZV and will help our understanding of the pathogenetic features of these emerging infections and allow us to evaluate new antiviral agents.

## Results

### Clinical findings in YEZV-infected mice

**Fig 1:**
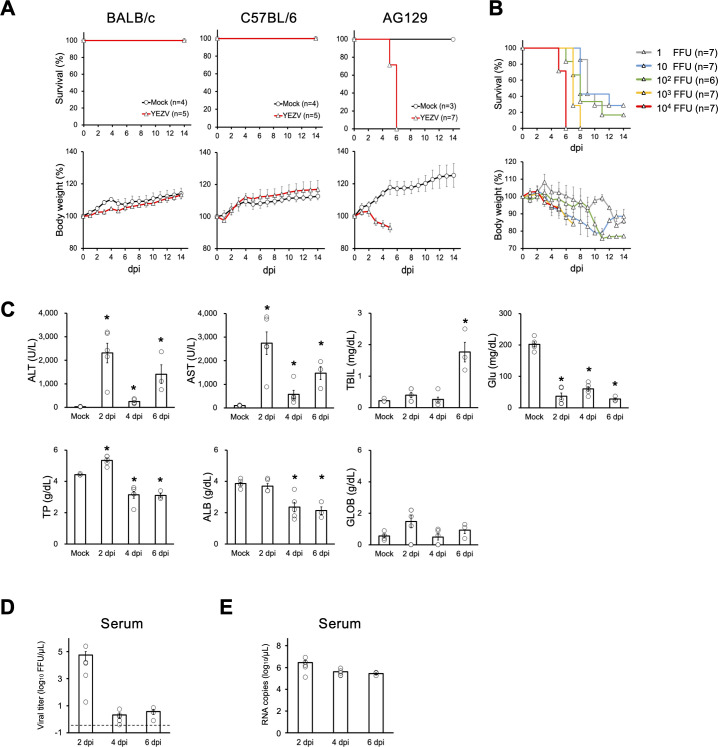
YEZV infection in mouse models. (A) Female 5-week-old C57BL/6 mice and BALB/c mice (n = 5) and sex-mixed 5-week-old AG129 mice (male: n = 4, female: n = 3) were inoculated intraperitoneally with 10^4^ FFU of YEZV. Fourteen-day survival rates and body weight changes were compared between mock and YEZV infected groups. Relative body weight is shown as means with standard deviations. (B) AG129 mice were inoculated intraperitoneally with 10^4^ FFU (male: n = 3, female: n = 3, red line), 10^3^ FFU (male: n = 4, female: n = 3, yellow line), 10^2^ FFU (male: n = 3, female: n = 3, green line), 10 FFU (male: n = 4, female: n = 3, blue line), and 1 FFU (male: n = 4, female: n = 3, gray line) of YEZV and monitored for 14-day survival rate and body weight change. Relative body weight was calculated and is shown as means with standard deviations. (C-E) AG129 mice were inoculated intraperitoneally with 10^4^ FFU of YEZV and sacrificed for serum sampling at 2, 4, and 6 dpi (2 dpi: n = 5, 4 dpi: n = 5, 6 dpi: n = 3) for (C) blood biochemistry tests and quantification of (D) viral titer and (E) viral RNA in serum. Means with standard errors were shown with individual values as white circles; (C) Alanine transaminase (ALT), aspartate transaminase (AST), total bilirubin (TBIL), total protein (TP), albumin (ALB), globlin (GLOB) and glucose (Glu) values in serum, (D) virus titers in serum quantified by focus-formation assay, and (E) copy numbers of viral RNA quantified by RT-qPCR. Statistical analyses were performed using the Steel test, and the significant difference (*p* < 0.05) is indicated as an asterisk. (D) Dotted line indicated the lower limit of detection (L.O.D.) for the assay (0.4 FFU/μL).

To confirm the pathogenicity of YEZV in mice, 10^4^ focus formation units (FFU) of YEZV isolated from a human patient were intraperitoneally inoculated into BALB/c, C57BL/6, and AG129 (type-I/II IFN receptor-knockout) mice [27]. Body weight and survival of the mice were monitored for 14 days after the viral challenge. The body weights of BALB/c and C57BL/6 mice inoculated with YEZV gradually increased, similar to mock-infected mice, for 14 days without any fatalities; however, the YEZV-inoculated AG129 mice showed body weight loss and 100% mortality by 6 days post-infection (dpi) (Fig 1A). Subsequently, AG129 mice were inoculated with serially diluted YEZV intraperitoneally or subcutaneously. The 14-day fatality rates for intraperitoneal inoculations of AG129 mice with 10^4^, 10^3^, 10^2^, 10 and 1 FFU of YEZV were 100%, 100%, 83%, 71% and 71%, respectively, with respective median survival rates of 6, 7, 8, 8 and 9 days (Fig 1B). Subcutaneous inoculation of AG129 mice with 10^4^, 10^3^ and 10^2^ FFU of YEZV resulted in 14-day fatality rates of 100%, 71% and 71%, respectively, with respective median survival rates of 8, 9 and 9 days (S1 Fig). Based on these results, an intraperitoneal inoculation of AG129 with 10^4^ FFU of YEZV, which will be 100% lethal, was used in all subsequent experiments as a lethal model of YEZV infection.

To investigate the pathogenesis of lethal YEZV infection in AG129 mice, AG129 mice intraperitoneally inoculated with 10^4^ FFU of YEZV were sacrificed for blood and organ collection at 2, 4, and 6 dpi. Blood biochemistry tests showed elevated levels of alanine transaminase (ALT), aspartate transaminase (AST), total bilirubin and decreased levels of total protein, albumin, and glucose in the YEZV-infected mice (Fig 1C). Marked elevation of ALT and AST levels were observed at 2 and 6 dpi, and interestingly, the increase in ALT and AST at 4 dpi was moderate. Serum total protein and albumin levels decreased significantly at 4 and 6 dpi, and blood glucose levels decreased remarkably at 2, 4, and 6 dpi (Fig 1C). Total bilirubin levels were found to be elevated only at 6 dpi (Fig 1C). Blood cell counts revealed increased white blood cells after 4 dpi (S2A Fig). White blood cell and platelet counts decreased slightly at 2 dpi, but there was no obvious leukopenia or thrombocytopenia as has been reported in human patients (S2A and S2B Fig). To examine the possibility of viremia, viral titers and viral RNA levels of YEZV in serum were quantified by focus formation assays and RT-quantitative PCR (RT-qPCR), respectively. Viral titers in serum peaked at 2 dpi and remarkably decreased after 4 dpi (Fig 1D). In contrast YEZV viral RNA in serum was detected at similar levels at 2, 4, and 6 dpi (Fig 1E).

### Pathological findings in YEZV-infected mice

**Fig 2:**
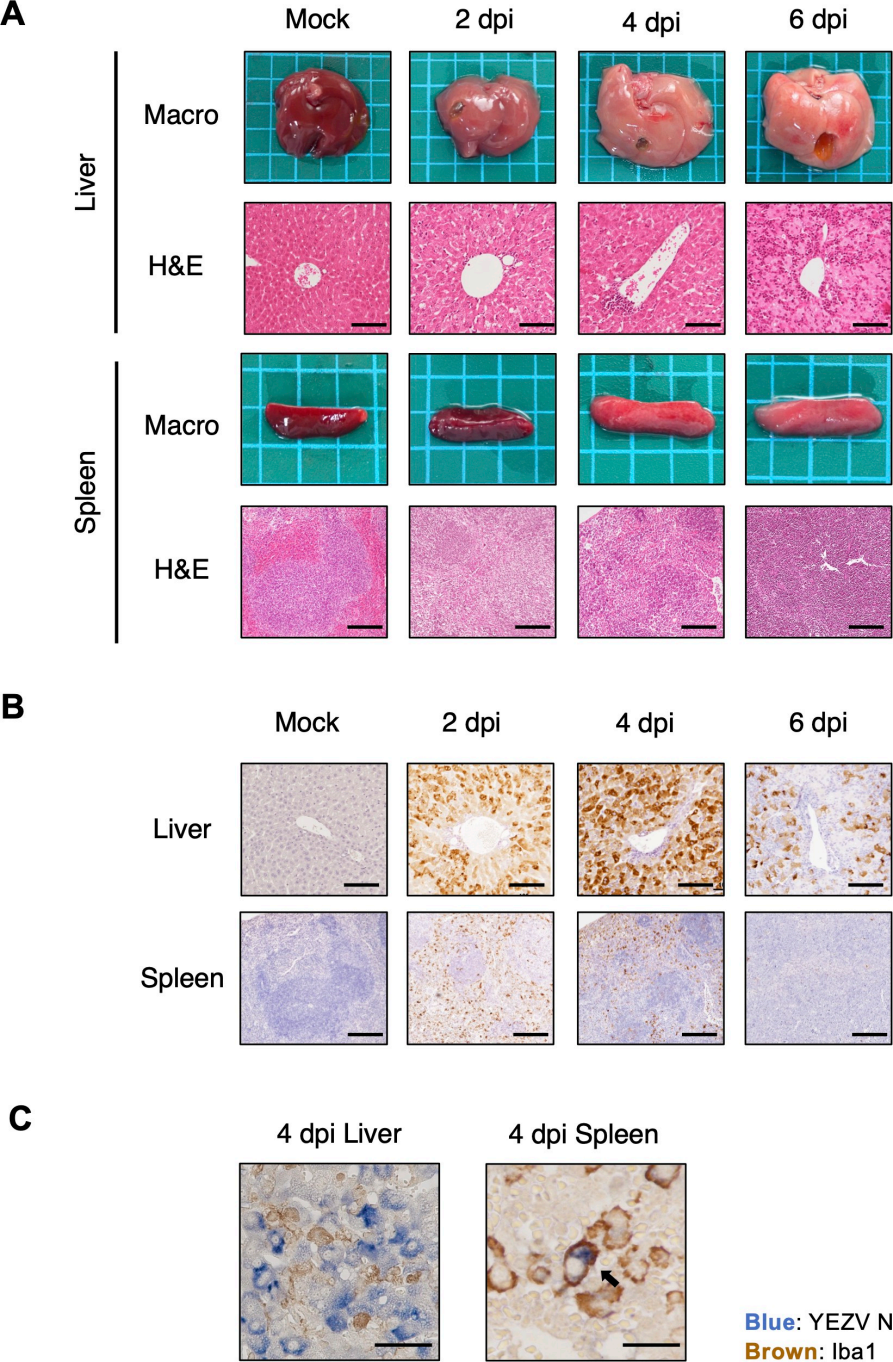
Histopathological findings in YEZV-infected AG129 mice. AG129 mice inoculated intraperitoneally with 10^4^ FFU of YEZV were sacrificed at 2, 4, and 6 dpi to collect organ samples. Mock-infected mice sacrificed at 6 dpi are shown as controls. Mouse organs were fixed in 10% phosphate-buffered formalin and (A) stained with hematoxylin-eosin (H&E) or (B and C) immunohistochemically stained for YEZV N. (A) Representative gross images and H&E-stained tissue images of the liver and spleen are shown. Each side of a square in the gross images is 5 mm. (B) Representative IHC images of the liver and spleen stained for YEZV N are shown with viral antigens in brown. (A and B) The scale bars on the histological images of the liver and spleen are 100 μm and 200 μm, respectively. (C) YEZV N protein (blue) and macrophage marker, Iba1 (brown) were stained by IHC double staining in the liver and spleen of YEZV-infected mice. The scale bars on IHC images of the liver and spleen tissues are 50 μm and 20 μm, respectively. Arrows indicate a cell in which both YEZV antigen and Iba1 were detected.

To identify the mechanism underlying fatal infection of YEZV in AG129 mice, histopathological analysis was conducted at 2, 4, and 6 dpi. Macroscopically, the livers and spleens of YEZV-infected AG129 mice were enlarged and whitish at 4 and 6 dpi compared to those of mock-infected mice (Fig 2A). Microscopically, the liver tissue structure was disrupted with hepatocyte degeneration and infiltration of inflammatory cells at 4 and 6 dpi (Fig 2A). The splenic follicular structures were obscured at 4 and 6 dpi (Fig 2A). No clear histological changes were observed in the kidney, heart or small intestine (S3 Fig). To characterize the distribution of the virus, YEZV antigens in the tissues were examined by immunohistochemical staining (IHC). Positive signals of YEZV antigen were diffusely observed in liver and spleen at 2 and 4 dpi and decreased at 6 dpi (Fig 2B). Viral antigens were detected mainly in hepatocytes in the liver, whereas Iba1(+) macrophages were positive for viral antigen in the spleen (Fig 2B and 2C). YEZV antigens were also detected in the kidney and heart, but at much lower levels than observed in the liver and spleen (S4 Fig). In contrast, in C57BL/6 and BALB/c mice intraperitoneally inoculated with 10^4^ FFU of YEZV, very few viral antigens were detected only in hepatocytes at 2 and 4 dpi (S5 Fig). Slight inflammatory cell infiltration was observed in the livers of these mice, but no clear pathological changes were observed in any of the organs including the liver (S6 Fig). We next examined whether the observed pathological changes in AG129 mice were dependent on the inoculation route. Similar pathological observations were also found in AG129 mice subcutaneously inoculated with YEZV (S7 and S8 Figs). These results suggest that YEZV replicates primarily in the liver and spleen and causes an acute hepatitis in AG129 mice.

**Fig 3:**
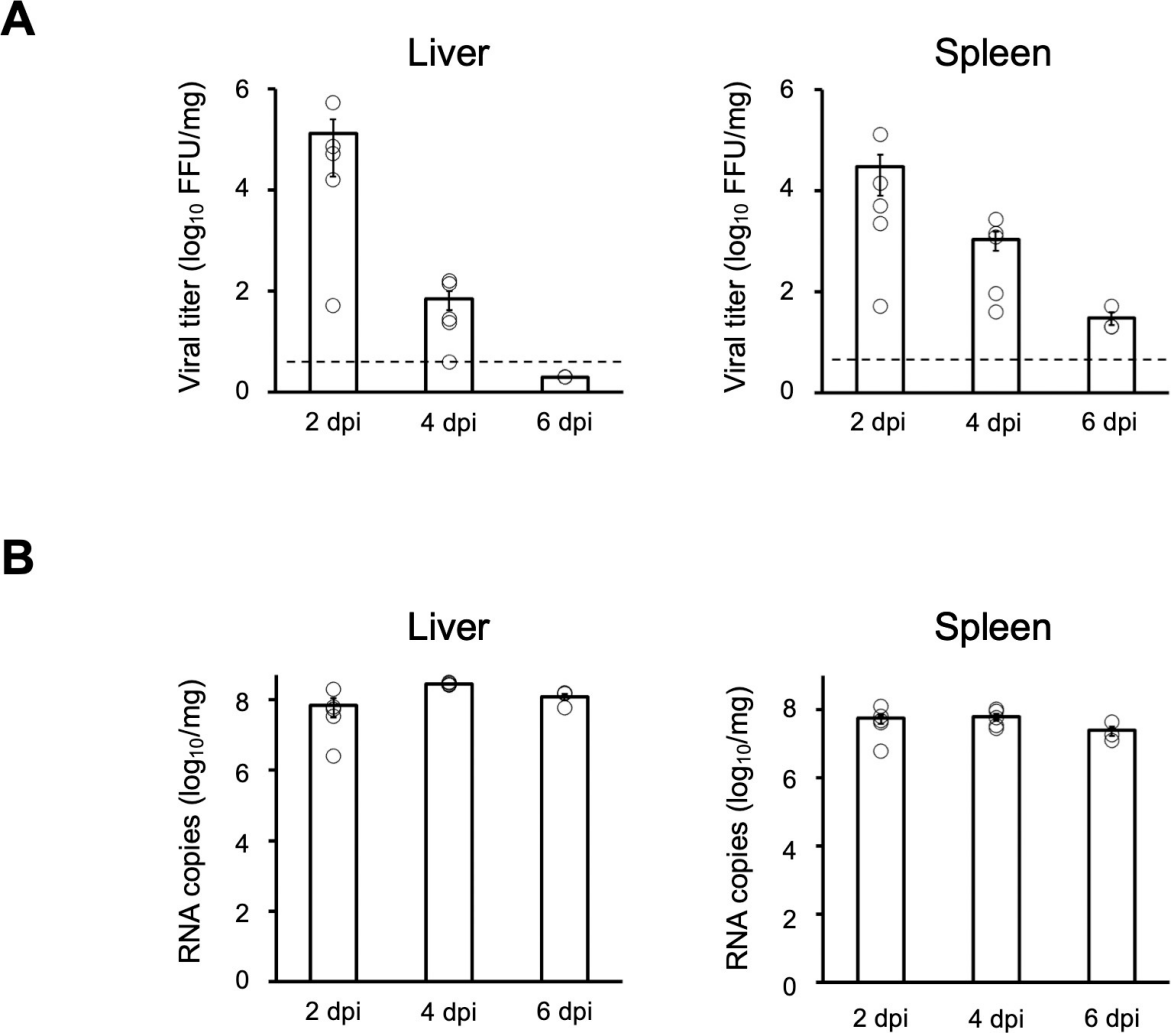
Viral titers and viral RNA in the liver and spleen of YEZV-infected AG129 mice. Livers and spleens of YEZV-inoculated AG129 mice collected at 2, 4 and 6 dpi were used (2 dpi: n = 5, 4 dpi: n = 5, 6 dpi: n = 3) for analyzing (A) viral titers quantified by focus-formation assay and (B) the copy numbers of viral RNA (YEZV L-segment) quantified by RT-qPCR. Dotted lines indicated the lower limit of detection (L.O.D.) for the assay (4 FFU/mg). Plots lower than the L.O.D. are indicated by the half value of L.O.D. on the graph. White circles, thick bars, and thin lines indicate individual values, mean values and standard errors, respectively.

To further confirm viral replication in the tissues, infectious virus and viral RNA loads were quantified. Viral titers in liver, spleen, kidney, heart peaked at 2 dpi and decreased in a time-dependent manner (Figs 3A and S9). Similarly, copy numbers of viral RNA were also detected in liver, spleen, kidney, and heart through 2 to 6 dpi (Figs 3B and S9). In contrast to the AG129 model, infectious virus particles were not detected in the serum, liver and spleen of either BALB/c and C57BL/6 mice at 2 dpi and 4 dpi, but viral RNA was detected in those samples (S10 Fig).

To rationalize the gap between the viral titer and the viral RNA copy number in the AG129 model, viral protein production in the liver was measured by Western Blotting. Consistent with the results of the IHC, the nucleoprotein (N) signal in the liver was not different between 2 dpi and 4 dpi (S11 Fig). On the other hand, at 4 dpi, the production of the envelope glycoprotein (Gn) was reduced, which may have resulted in an inability to form complete viral particles and a concomitant reduction of viral titer (S11 Fig).

## Verification of the anti-YEZV effects of T-705 in the YEZV-infected AG129 mouse model

**Fig 4:**
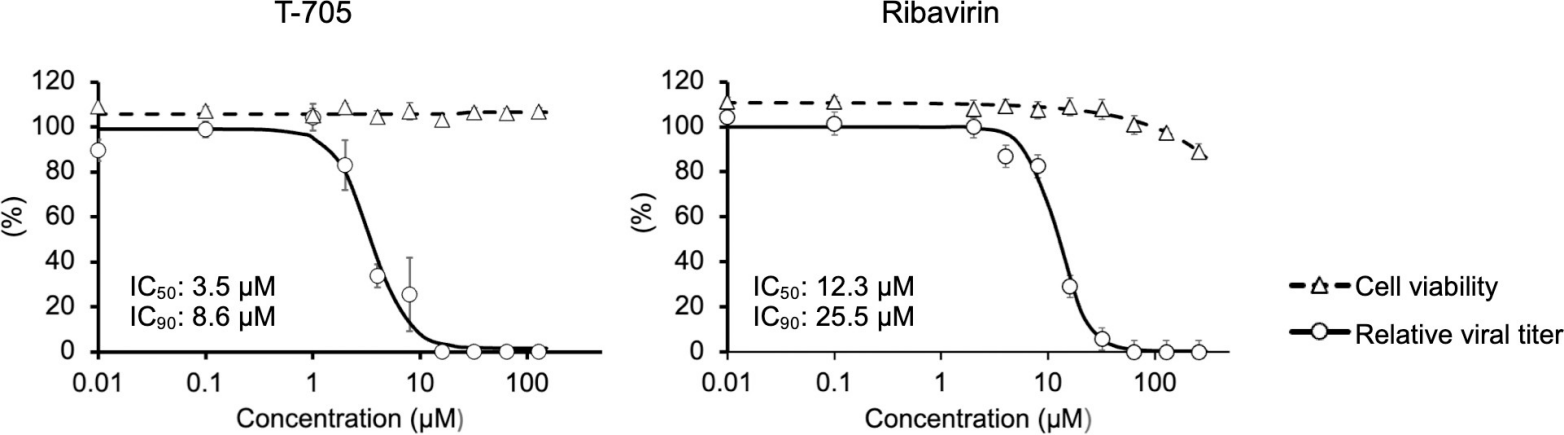
The effects of antivirals on YEZV infection *in vitro*. Inhibitory effects of T-705 and ribavirin on YEZV replication in Vero cells were verified. Viral titers (white circles and solid lines) in the supernatant and cell viability (white triangles and dotted lines) at 6 dpi were compared with those in the absence of the chemical compounds.

The nucleic acid analogs, T-705 and ribavirin have been shown to have antiviral effects against a broad spectrum of RNA viruses [28,29], including human pathogenic tick-born bunyaviruses, severe fever with thrombocytopenia syndrome virus (SFTSV) and CCHFV [30–33]. The anti-YEZV effects of T-705 and ribavirin were evaluated using Vero cells *in vitro*. Both T-705 and ribavirin inhibited YEZV replication in Vero cells (Fig 4). The 50% and 90% inhibitory concentrations (IC_50_ and IC_90_, respectively) of T-705 were 3.51 μM and 8.79 μM, respectively, and those of ribavirin were 12.32 μM and 25.47 μM, respectively. Since T-705 showed a stronger antiviral effect than ribavirin *in vitro*, the antiviral effect of T-705 was further analyzed *in vivo* using our established animal model.

**Fig 5:**
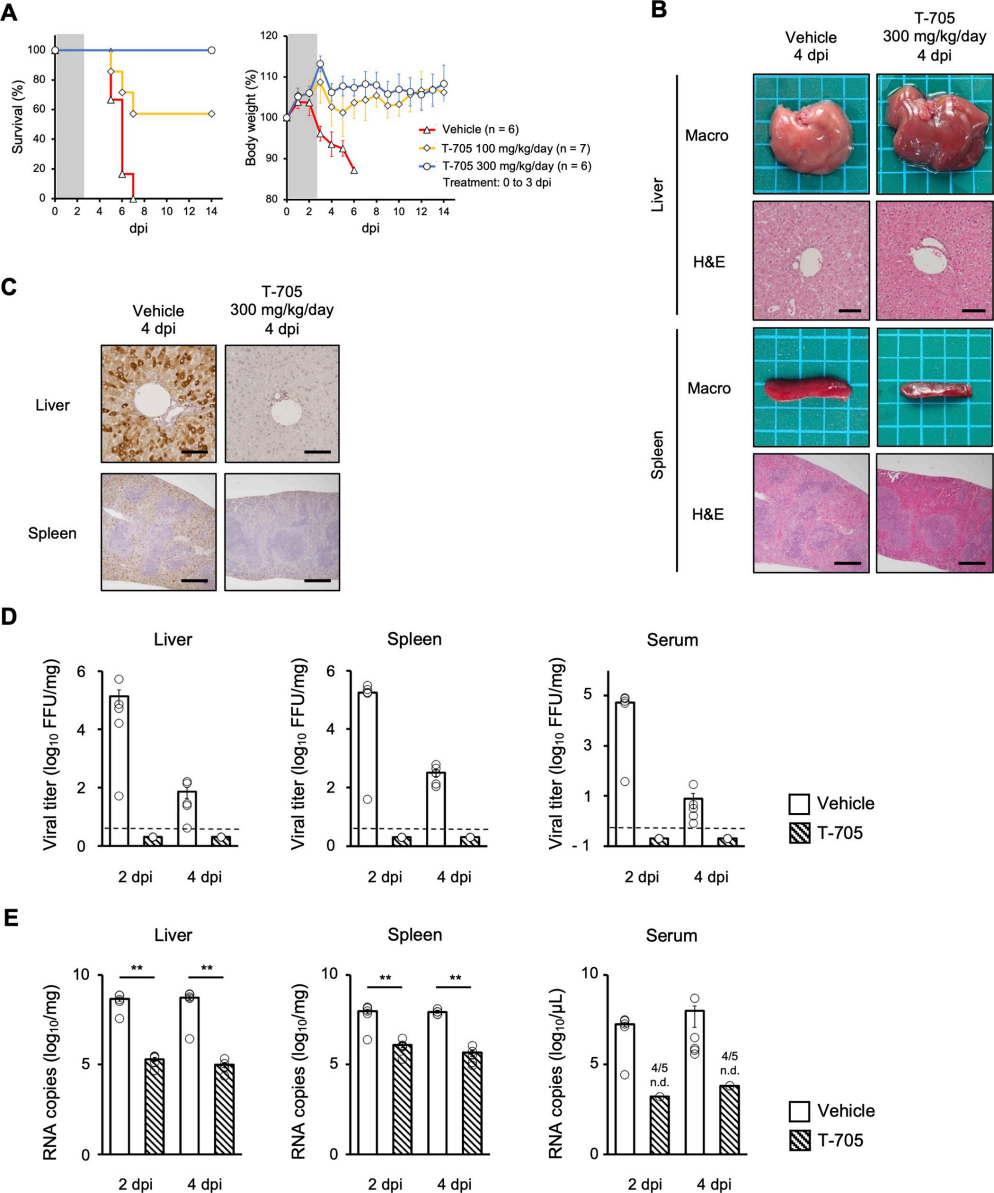
Effects of antivirals on YEZV infection *in vivo*. (A) The antiviral effects of T-705 in the AG129 mouse model were evaluated. AG129 mice intraperitoneally inoculated with 10^4^ FFU of YEZV were orally dosed with T-705 (100 or 300 mg/kg/day) or vehicle using a stomach probe at 1 hour, 1, 2 and 3 dpi (represented by gray color on the figure). All mice were monitored for survival (left panel) and body weight changes (right panel) for 14 days. Fourteen-day survival rates and body weight changes were compared with the vehicle-treated group, 100 mg/kg/day of T-705-treated group and 300 mg/kg/day of T-705-treated group using a log-rank test. (B-E) Vehicle- or 300 mg/kg/day of T705-treated YEZV-infected AG129 mice were sacrificed at 2 and 4 dpi to collect blood and tissue samples. (B) Representative gross images and H&E-stained tissue images of the liver and spleen of mice are shown. Each side of a square in the gross images is 5 mm. The scale bars on the histological images of the liver and spleen are 100 μm and 500 μm respectively. (C) YEZV N proteins were detected by IHC in the liver and spleen of vehicle-treated and T-705-treated mice, and representative images are shown. The scale bars on the images of the liver and spleen tissues are 100 μm and 500 μm, respectively. (D) Viral titers and (E) the copy number of viral RNA (YEZV L-segment) in the liver and spleen of vehicle-treated and T-705-treated mice were quantified. Dotted lines indicated the lower limit of detection (L.O. D.) for the assay (4 FFU/mg of liver and spleen and 0.4 FFU/μL of serum). Plots lower than the L.O.D. are indicated by the half value of L.O.D. on the graph. Statistical differences in viral RNA copy number between the vehicle-treated group and T-705-group were examined by the Mann-Whitney U test, and the significant difference (*p* < 0.01) is indicated as double asterisks.

To evaluate this model for the investigation of antiviral agents, YEZV-infected AG129 mice were treated with 100 mg/kg or 300 mg/kg of T-705 immediately after the viral challenge. In these experiments, T-705 was orally administered once a day from 1 hour post-infection to 3 dpi. All mice in the vehicle-treated group died up to 7 dpi with weight loss (Fig 5A). In contrast 100 mg/kg/day and 300 mg/kg/day of T-705-treated groups showed 57% and 100% survival rates respectively (Fig 5A). Mice in the T-705-treated group which survived during the monitoring period showed milder body weight loss than those in the vehicle-treated group (Fig 5A).

To investigate the effects of T-705 treatment on tissue damage and viral replication, liver, spleen, and serum were collected at 2 and 4 dpi for pathological and virological analysis. Hepatocyte degeneration and infiltration of inflammatory cells in the liver was milder in T-705-treated mice than vehicle-treated mice (Fig 5B). The follicular structure of the spleen was preserved at 4 dpi in T-705-treated mice (Fig 5B). Positive signals for YEZV N proteins in the liver and spleen were markedly less in T-705-treated mice than in vehicle-treated mice (Fig 5C). Viral titers in liver, spleen, and serum of T-705-treated mice were under the detection limits (Fig 5D). Consistent with the virus antigen expression and titer, the copy number of YEZV viral RNA in liver, spleen and serum of T-705-treated mice was significantly lower than that of vehicle-treated mice (Fig 5E). In summary, the mouse model of YEZV infection established in this study clearly demonstrated the anti-YEZV effect of T-705 *in vivo*.

## Discussion

YEZV is an emerging tick-borne orthonairovirus belonging to the Sulina genogroup and the first human pathogenic orthonairovirus in the Sulina genogroup. Together with the Tamdy genogroup viruses, which are phylogenetically related to the Sulina genogroup, YEZV can be considered as an etiological agent of emerging orthonairovirus infections in East Asia and thus, has the potential to be a burden on public health [8–11]. However, no pathological analyses of the orthonairoviruses belonging to the Sulina and Tamdy genogroups have been conducted. Our mouse model for YEZV infection is the first for emerging tick-borne orthonairoviruses involving the Sulina and Tamdy genogroup viruses.

Previous studies have used type-I interferon receptor-knockout mice with either C57BL/6 or 129 backbone, STAT-1 knockout mice and mice treated with cyclophosphamide to establish infection models of human pathogenic orthonairoviruses such as CCHFV and Dugbe virus [14–16]. Here, we have demonstrated that AG129 mice, which are widely utilized in the field of flavivirus research as lethal infection models [34–38], can also be utilized for establishing a model of tick-borne orthonairovirus infections. Because YEZV was isolated from a human patient using AG129 mice that showed swelling of the spleen [12], AG129 mice was our first choice for establishing a mouse model of YEZV infection.

In the present study, we have characterized in detail the pathogenesis of lethal infection of YEZV in AG129 mice. Consistent with previous reports of the other human pathogenic orthonairoviruses, YEZV was lethal in immunodeficient but not in wild-type mice. Wild-type mice did not show any clinical symptoms but potentially developed viremia. Therefore, they may be important to utilize them as a relatively mild disease model in future studies. In comparison to the immunodeficient mouse models of orthonairoviruses, such as CCHFV, Hazara virus, and Tofla virus models, whose LD_50_ were usually below 10 FFU or plaque forming unit regardless of the inoculation route, the LD_50_ of YEZV infection in AG129 mice was compatible [16,17,19,21]. YEZV replicated mainly in the liver and spleen in AG129 mice, resulting in severe liver inflammation. Infectious virus was rapidly eliminated before the AG129 mice died, but the severe hepatitis remained even after virus clearance and could have contributed to the observed mortality. Elevated liver enzyme levels, including AST and ALT, consistent with reported human cases [12,13], also suggests serious liver damage as found in mouse models of other orthonairovirus infection, including CCHFV, Hazara virus, Leopards Hill virus, and Keterah virus [15–18,20–22,39]. Interestingly, the present mouse model showed a biphasic elevation of AST and ALT value consistent with biphasic-like increase of AST in a human patient [12], and which has not been reported in the other orthonairovirus infections in humans and also animal models. In the studies of CCHFV, Kupffer cells, hepatocytes, and endothelial cells have been identified as the primary target cells in mouse and macaque infection models and in human patients [2,15,20,40–44]. Kupffer cells have been proposed to be important for viral spread and replication in the liver during the early stages of CCHFV infection [20,41,42]. However, in YEZV-infected mice, most of the virus antigen-positive cells in the liver were hepatocytes and only a small number of virus antigen-positive Kupffer cells were detected, even though virus antigen-positive signals were detected in Iba1(+) macrophages in the spleen. While infectious viral particles and/or virus RNAs were detected in the kidneys and heart, no tissue damage or severe inflammation was observed histopathologically. In addition, blood urea nitrogen and creatinine levels, indicators of renal function, were normal. Gastrointestinal disturbances as reported in mouse models of Leopards hill virus and Tofla virus [19,22] were not observed. These results suggest that YEZV is a hepatotropic virus like the other orthonairoviruses, although the less tissue damage other than in hepatocytes might be one of the unique features of YEZV that distinguishes it from the other orthonairoviruses, such as CCHFV.

In this model, even though the viral N was detected in the liver at 2 dpi and 4 dpi by both IHC and immunoblot analysis, the viral titer at 4 dpi was significantly lower than that at 2 dpi. On the other hand, the expression of viral Gn was significantly reduced at 4 dpi compared to 2 dpi. Therefore, defective expression of the glycoprotein could be an additional factor limiting virus elimination. Furthermore, although viral titers in tissue and serum declined after 4 dpi, the copy numbers of viral RNA in tissue and serum remained stable until 6 dpi. Together with the temporal reduction of AST and ALT levels at 4 dpi, later liver injury could be caused without the production of infectious virus particles but may still be associated with RNA replication.

Since there are not currently active therapeutic agents for treatment of these newly emerging tick-borne orthoanirovirus infections, discovery of new antiviral agents are required. In this study, the anti-YEZV effects of T-705 and ribavirin were evaluated, and both agents inhibited YEZV replication *in vitro*. Consistent with other human pathogenic tick-borne bunyaviruses including CCHFV and SFTSV, T-705 showed smaller IC_50_ and IC_90_ than ribavirin against YEZV *in vitro* [30,32,45]. In addition to our *in vitro* results, since T-705 showed better therapeutic effect than ribavirin in mouse infection models of CCHFV, SFTSV, and HRTV [30,33,46], we evaluated the anti-viral effect of T-705 using the YEZV infection mouse model. Oral administration of T-705 rescued YEZV-infected AG129 mice from lethal infection in a dose-dependent manner, and viral titers in tissues of T-705-treated mice were lower than the limit of detection. In addition, severe inflammation was not observed in tissues of mice treated with T-705. These results suggest that T-705 inhibited YEZV replication in the early stages of infection, thus preventing lethal inflammation.

Recently reported novel pathogenic tick-borne orthonairovirus infections associated with the Tamdy and Sulina genogroups [8–13] have not been fully analyzed for their pathogenesis in humans as patients have been mainly identified by retrospective studies. Analysis using the present mouse model will contribute to our understanding of viral pathogenesis. In the future, it will be important to conduct detailed analyses in both patients and animal models to identify common pathological features. It will also be important to consider co-infections with other pathogens with tick-borne virus, which has been recently highlighted [47]. In fact, four of the seven YEZV-infected patients reported in Japan were infected with *Borrelia* spp. [12]. Furthermore, symptoms such as fever and malaise seen in YEZV-infected patients are atypical and might be difficult to distinguish from other infections. A co-infection mouse model of tick-borne pathogens may facilitate the development of broad-spectrum pan-tick-borne disease treatment.

## Materials and methods

### Ethics statement

All animal experiments were approved by the Animal Care and Use Committee of Hokkaido University (Approval numbers: 18–0149 and 23–0063) and were performed in accordance with the committee’s guidelines.

### Cells and viruses

Vero (JCRB9013) and Vero E6 (JCRB9007) cells were maintained in Dulbecco’s Modified Eagle’s Medium (DMEM) supplemented with 10% fetal bovine serum (FBS), 100 unit/mL penicillin, and 100 μg/mL streptomycin at 37°C with 5% CO_2_.

YEZV strain HH003-2019 was isolated from infected patients as previously reported [12]. Briefly, the virus was isolated from the supernatant of Vero E6 cells inoculated the AG129 mouse serum collected at 5 days after intraperitoneally inoculation of infected patient’s plasma. The virus was stored at -80°C until use.

### Mouse infection experiments

All animal experiments were conducted in a BSL-2 laboratory at the International Institute for Zoonosis Control, Hokkaido University with the above-described ethical approval. Female 5-week-old BALB/cCrSlc and C57BL/6NCrSlc mice were obtained from Japan SLC and used for animal experiments. AG129 mice, double knockout immunocompromised mice lacking both type I and type II interferon receptors (S129 background), were obtained from Marshall BioResources and maintained in our laboratory. Sex-mixed 5-weeks-old AG129 mice were used for the animal infection experiments.

BALB/cCrSlc, C57BL/6NCrSlc and AG129 mice were intraperitoneally inoculated with 100 μL of DMEM with or without 10^4^ FFU of YEZV under anesthesia. All mice were monitored for body weight changes and survival for 14 days, and in order to collect blood and tissue samples, five mice were mock infected or infected with YEZV and sacrificed at 2, 4, and 6 days post-infection (dpi), respectively. A portion of the harvested tissues was homogenized in DMEM to prepare a 10% tissue emulsion for virus titration and RNA extraction. The other tissues were stored in phosphate-buffer with 10% formalin for histopathological analyses. Blood biochemistry tests were conducted using VetScan VS2 with Multi Roter I Preventive Care Panel (Zoetis Japan, Tokyo, Japan). For determining LD_50_, AG129 mice were intraperitoneally or subcutaneously inoculated with 100 μL of DMEM with or without 10^4^, 10^3^, 10^2^, 10 or 1 FFU of YEZV under anesthesia and observed daily for body weight changes and survival for 14 days.

### Virus titration

The titers of virus used in this study were defined as FFU. The samples diluted in DMEM were inoculated into Vero cells on 24-well plates and incubated at 37°C for 1 hour. The supernatants were removed, and the treated cells were cultured in Minimum Essential Medium (MEM) containing 1% methylcellulose and 2% FBS for 6 days. The cells were then fixed with methanol for 20 minutes at –30°C. After blocking with PBS containing 1% bovine serum albumin for 30 minutes, the cells were incubated with 1,000-time-diluted YEZV-infected mouse serum for 1 hour. After washing three times with PBS, the cells were incubated with Goat Anti-Mouse IgG H&L-HRP (1:1,000, Abcam, Cambridge, U.K.) for 1 hour. After washing in the same manner, the cells were incubated with 3,3’-Diaminobenzidine (DAB), tetrahydrochloride (Nacalai twsque, Kyoto, Japan) for 10 to 20 minutes. Stained foci were counted, and the titers were determined. The staining procedure was performed at room temperature.

### RNA extraction and Quantitative real-time PCR

Total RNA was extracted from the 10% tissue emulsions, mice serum or cell culture supernatant using TRIzol LS Reagent (Thermo Fisher Scientific, U.S.) and used to quantify YEZV viral RNA expression. Quantities of YEZV viral RNA were measured using RT-qPCR with One Step PrimeScript III RT-qPCR Mix (Takara Bio, Shiga, Japan) and the specific primers (5’-GGTGTAAAGCCCAACATCCT-3’ and 5’-CTCAACCTGCTTCCAACCTATC-3’) and probe (5’-/5Cy5/CCAAGGAAG/TAO/CACACAGATGGGTACA/3IAbRQSp/-3’) for the L gene. The RT-qPCR reaction protocol included 5 minutes at 52°C, 10 seconds at 95°C and then 40 cycles of 5 seconds at 95°C and 30 seconds at 60°C. The signals were measured at the elongation step. A cloned plasmid was used for the standard to estimate the copy number. RT-qPCR was performed using qTower^3^ G (Analytik Jena, Jena, Germany), and the obtained data were analyzed by qPCRsoft (Analytik Jena, Jena, Germany).

### Histopathological analysis

Formalin-fixed, paraffin embedded tissues were sectioned into 3 μm and stained with hematoxylin-eosin or used for immunohistochemical staining (IHC). IHC was performed using YEZV-N peptide (POS: 165–182) immunized rabbit serum (1:20,000) (Cosmo Bio, Tokyo, Japan) or anti Iba1 goat polyclonal antibody (1:500, #001–27991, Wako, Osaka, Japan) as primary antibodies. For YEZV antigen detection, peroxidase-labeled goat anti-rabbit IgG polyclonal antibody (Nichirei Bioscience, Tokyo, Japan) and DAB were used as secondary antibodies and substrates, respectively. In the Iba1 and YEZV antigen double staining experiments, ImmPRESS (Peroxidase) Polymer Anti-Goat IgG Reagent (Vector laboratories, California, USA) and alkaline phosphatase-labeled goat anti-rabbit IgG polyclonal antibody (Nichirei Bioscience, Tokyo, Japan) were used as secondary antibodies and ImmPACT AMEC Red Peroxidase Substrate (Vector laboratories, California, USA) and Vector Blue (Vector laboratories, California, USA) as substrates. All images were acquired using Nikon ECLIPSE 80i light microscopy (Nikon, Tokyo, Japan).

### Western blotting

Liver emulsions, YEZV-N protein transfected 293T cells and YEZV-G protein transfected HEK293 cells were treated with an EzRipa Lysis Kit (ATTO, Tokyo, Japan). The treated samples were separated by SDS-PAGE and subsequently transferred onto polyvinylidene difluoride membranes (Merck Millipore, Burlington, MA). The membranes were blocked for 1 hour with 0.05% Tween 20 in TBS (TBST) that contained 5% skim milk at 25°C. The membranes were incubated overnight at 4°C with YEZV-N peptide immunized rabbit serum (1:1,000) or YEZV-G peptide (POS: 349–369) immunized rabbit serum (1:1,000) (Cosmo Bio, Tokyo, Japan) in TBST with 0.05% skim milk. After washing with TBST, the membranes were incubated 1 hour at 25°C with Goat Anti-Rabbit IgG H&L-HRP (1:10,000, Abcam) in TBST with 0.05% skim milk. The membranes were washed again with TBST and subsequently incubated with Immobilon Western Chemiluminescent HRP Substrate (Merck Millipore) to visualize the peroxidase signal. Beta-actin was detected by the same protocol using Anti-β-Actin pAb-HRP-DirecT (MEDICAL & BIOLOGICAL LABORATORIES CO., LTD., Tokyo, Japan) and HRP substrates. Chemical luminescent signals were detected by Amersham imageQuant 800 (Cytiva, Massachusetts, USA) and signal intensities were quantified using ImageJ software [48].

### Verification of the efficacy of antiviral agents

Favipiravir (T-705) (Angene International, Nanjing, China) and ribavirin (Fujifilm Wako Pure Chemical, Osaka, Japan) was purchased from Namiki Shoji (Tokyo, Japan) and Fujifilm Wako Pure Chemical Industries, respectively. T-705 was dissolved in dimethyl sulfoxide (DMSO) for *in vitro* experiments and in 0.5% methyl cellulose 400 solution for *in vivo* experiments. Ribavirin was dissolved in DMSO for *in vitro* experiments.

In the cultured cell infection experiments, Vero cells were infected with YEZV at a multiplicity of infection (MOI) of 0.001 per cell in the presence of various concentration of T-705 (0.01, 0.1, 1, 2, 4, 8, 16, 32, 64, 128 μM) or ribavirin (0.01, 0.1, 2, 4, 8, 16, 32, 64, 128, 256 μM). Supernatants of cell culture were then collected at 6 dpi and used for virus titration. The 50% inhibitory concentration (IC_50_) and the 90% inhibitory concentration (IC_90_) were calculated by regression analysis using ImageJ software [48]. The MTT assay was performed to evaluate cell viability according to previously reported methods [49]. Vero cells were cultured for 6 days in the presence of the designated concentration of agents without viral infection. Cell viability was calculated as follows: [(absorbance of cell culture supernatant in the presence of the agent − absorbance of culture medium without cells in the absence of the agent)/(absorbance of cell culture supernatant in the absence of the agent − absorbance of culture medium without cells in the absence of the agent)] × 100. Viral replication reduction assays and cell viability assays were performed in triplicate.

In the animal experiments, each AG129 mouse was intraperitoneally inoculated with 100 μL of YEZV (10^4^ FFU). The infected mice were orally dosed with T-705 (100 or 300 mg/kg/day) or vehicle using a stomach probe at 1 hour, 1 day, 2 days and 3 days post-infection. The body weight change and survival of 6 vehicle-treated-mice, 7 mice treated with 100 mg/kg/day of T-705 and 6 mice treated with 300 mg/kg/day of T-705 were monitored for 14 days. In order to collect blood and tissue samples, 5 mice treated with 300 mg/kg/day T-705 and 5 mice treated with vehicle were sacrificed at 2 and 4 dpi, respectively. Viral titration, RT-qPCR analysis and histopathological analysis were conducted as described above.

### Statistical analysis

Survival rates were compared using log-rank test. Blood biochemistry levels were compared between uninfected and infected mice by the steel test. Statistical differences in viral RNA copy number between the two groups were examined by the Mann-Whitney U test. All statistical analyses were performed using EZR [50]. Statistical significance was set at *P* < 0.05.

## Supporting information

S1 Fig Survival rate and body weight change of AG129 mice subcutaneously inoculated with YEZV. AG129 mice were inoculated subcutaneously with 10^4^ FFU (male: n = 3, female: n = 4, red line), 10^3^ FFU (male: n = 3, female: n = 4, yellow line), and 10^2^ FFU (male: n = 4, female: n = 3, green line) of YEZV and monitored the 14-day survival and body weight change. Relative body weights are shown as means with standard deviations.
(TIF)

S2 Fig Hematological tests in YEZV-infected AG129 mice. AG129 mice were inoculated intraperitoneally with 10^4^ FFU of YEZV and sacrificed for serum sampling at 2, 4, and 6 dpi (2 dpi: n = 5, 4 dpi: n = 5, 6 dpi: n = 3) and blood cell counting. The number of (A) white blood cells (WBC) and (B) platelets (PLT) are indicated. White circles, thick bars, and thin lines indicate individual values, mean values and standard errors, respectively. Statistical analyses were performed using the Steel test; *p < 0.05.
(TIFF)

S3 Fig Representative hematoxylin-eosin-stained tissue images of the kidney, heart and small intestine of mock and YEZV-inoculated AG129 mice at 4 dpi. Representative H&E-stained histological images of the kidney, heart and small intestine of mock and YEZV-inoculated mice at 4dpi are shown. The scale bars on the histological images are 100 μm.
(TIFF)

S4 Fig Representative IHC images of the kidney and heart of mock and YEZV-inoculated AG129 mice. Representative IHC images of the kidney and heart of mock and YEZV-inoculated mice at 4dpi are shown. Viral antigens were detected using anti-YEZV N protein rabbit antibody. The scale bars on the histological images are 100 μm.
(TIFF)

S5 Fig Representative IHC images of the liver of YEZV-inoculated BALB/c mice and C57BL/6 mice. Representative IHC images of the liver of YEZV-inoculated BALB/c mice and C57BL/6 mice at 2 and 4dpi are shown. Viral antigens were detected using anti-YEZV N protein rabbit antibody. Arrows indicate viral antigen-positive cells. The scale bars on the histological images are 200 μm.
(TIFF)

S6 Fig Representative hematoxylin-eosin-stained tissue images of the liver of YEZV-inoculated BALB/c mice and C57BL/6 mice at 4 dpi. Representative H&E-stained histological images of the liver of YEZV-inoculated BALB/c mice and C57BL/6 mice at 2 and 4 dpi are shown. The scale bars on the histological images are 200 μm.
(TIFF)

S7 Fig Representative hematoxylin-eosin-stained tissue images of the liver and spleen of AG129 mice subcutaneously inoculated with YEZV. AG129 mice were subcutaneously inoculated with 10^4^ FFU of YEZV and sacrificed for organs sampling at 3 and 5 dpi. Representative H&E-stained histological images of the liver and spleen of the mice at 3 and 5 dpi are shown. The scale bars on the histological images are 200 μm.
(TIFF)

S8 Fig Representative IHC images of the liver and spleen of AG129 mice subcutaneously inoculated with YEZV. AG129 mice were subcutaneously inoculated with 10^4^ FFU of YEZV and sacrificed for organs sampling at 3 and 5 dpi. Viral antigens were detected using anti-YEZV N protein rabbit antibody. The scale bars on the histological images are 200 μm.
(TIFF)

S9 Fig Viral titers and viral RNA in the kidney and heart of YEZV-infected AG129 mice. Kidneys and hearts of YEZV-inoculated AG129 mice collected at 2, 4 and 6 dpi were crushed to prepare emulsions (2 dpi: n = 5, 4 dpi: n = 5, 6 dpi: n = 3). Virus titers and viral RNA (YEZV L segment) were quantified by focus-formation assays and RT-qPCR respectively. Plots lower than the limit of detection (L.O.D.) of viral titration (4 FFU/mg) are indicated by the half value of L.O.D. on the graph. White circles, thick bars, and thin lines indicate individual values, mean values and standard errors, respectively.
(TIF)

S10 Fig Viral RNA load of YEZV-infected BALB/c and C57BL/6 mice. Serum, livers, and spleens of YEZV-inoculated BALB/c and C57BL/6 mice were collected at 2 and 4 dpi (2 dpi: n = 3, 4 dpi: n = 3) and subjected to RNA extraction. The amount of viral RNA (YEZV L segment) was quantified by RT-qPCR. White circles, thick bars, and thin lines indicate individual values, mean values, and standard errors, respectively.
(TIF)

S11 Fig Quantification of YEZV-G and N protein expression in the liver of YEZV infected AG129 mice. (A and B) Equal amounts (4 μg) of mock-infected and YEZV-infected mice liver emulsions were separated by SDS -PAGE. Immunoblotting analyses were conducted using anti-YEZV Gn, anti-YEZV N and anti-beta-actin antibodies. HEK 293 cells transfected with YEZV Gn or YEZV N were used as positive controls. (B) Signal intensities were quantified using ImageJ software. White circles, thick bars, and thin lines indicate individual values, mean values and standard errors, respectively.
(TIF)

S1 Data Data that underlies this paper. All data used to build the graphs are available in this file.
(XLSX)
