# Supplementary figures and images for: Establishment of a lethal mouse model of emerging tick-borne orthonairovirus infections
Source: PLoS Pathog. 2024 Mar 19;20(3):e1012101. doi: 10.1371/journal.ppat.1012101 (PMC10980201; doi:10.1371/journal.ppat.1012101)

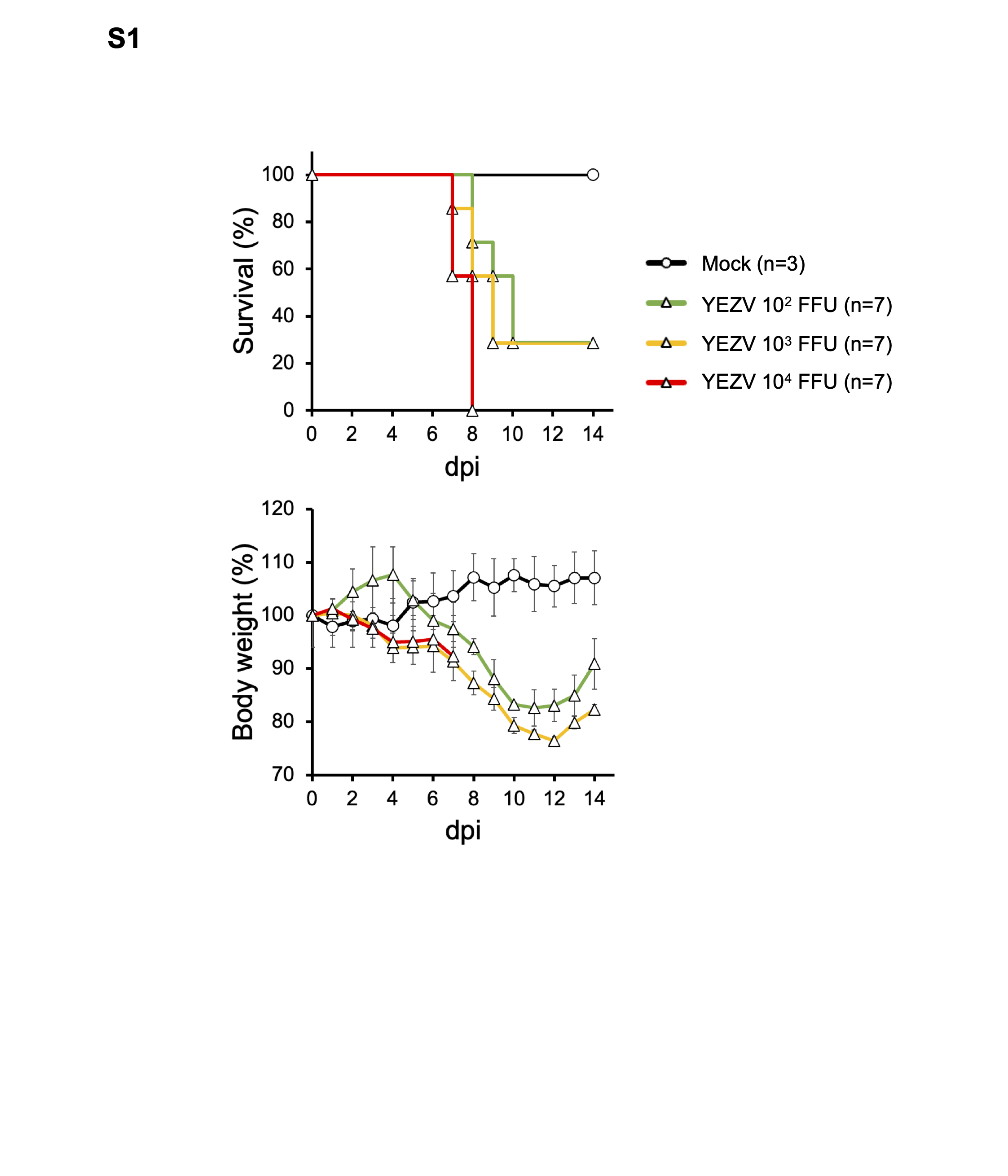

Supplement: S1 Fig — AG129 mice were inoculated subcutaneously with 104 FFU (male: n = 3, female: n = 4, red line), 103 FFU (male: n = 3, female: n = 4, yellow line), and 102 FFU (male: n = 4, female: n = 3, green line) of YEZV and monitored the 14-day survival and body weight change. Relative body weights are shown as means with standard deviations. (TIF) [file ppat.1012101.s001.tif]

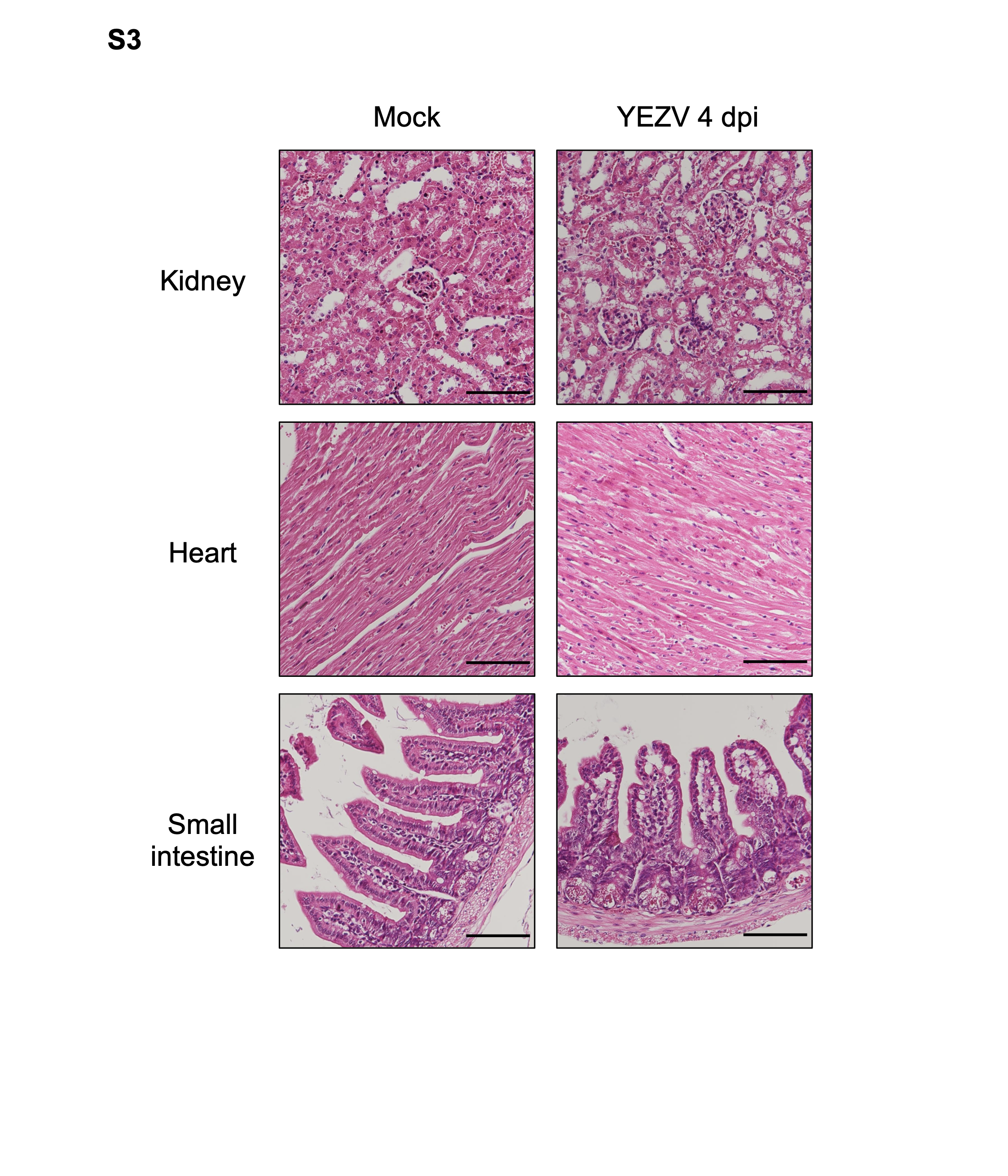

Supplement: S3 Fig — Representative H&E-stained histological images of the kidney, heart and small intestine of mock and YEZV-inoculated mice at 4dpi are shown. The scale bars on the histological images are 100 μm. (TIFF) [file ppat.1012101.s003.tiff]

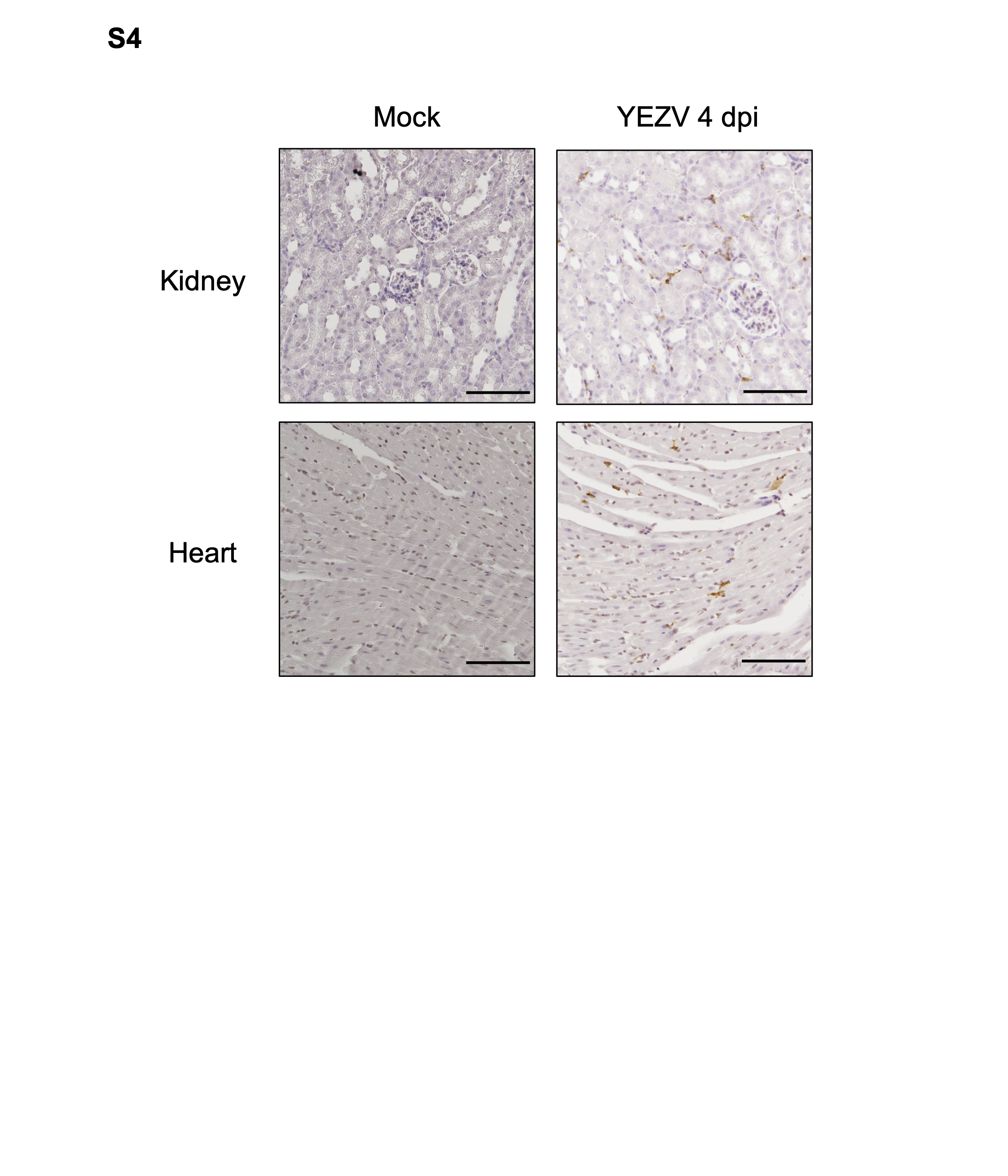

Supplement: S4 Fig — Representative IHC images of the kidney and heart of mock and YEZV-inoculated mice at 4dpi are shown. Viral antigens were detected using anti-YEZV N protein rabbit antibody. The scale bars on the histological images are 100 μm. (TIFF) [file ppat.1012101.s004.tiff]

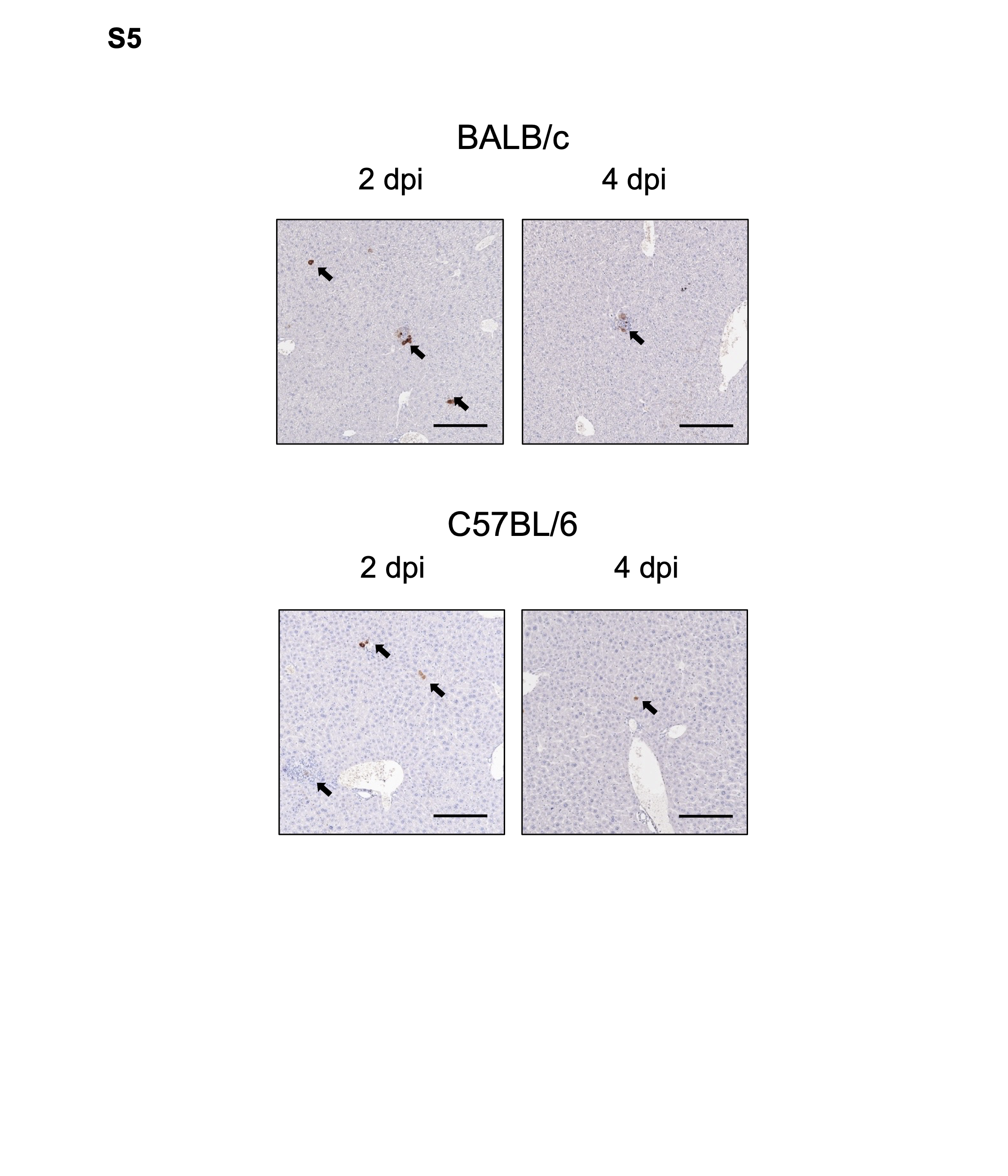

Supplement: S5 Fig — Representative IHC images of the liver of YEZV-inoculated BALB/c mice and C57BL/6 mice at 2 and 4dpi are shown. Viral antigens were detected using anti-YEZV N protein rabbit antibody. Arrows indicate viral antigen-positive cells. The scale bars on the histological images are 200 μm. (TIFF) [file ppat.1012101.s005.tiff]

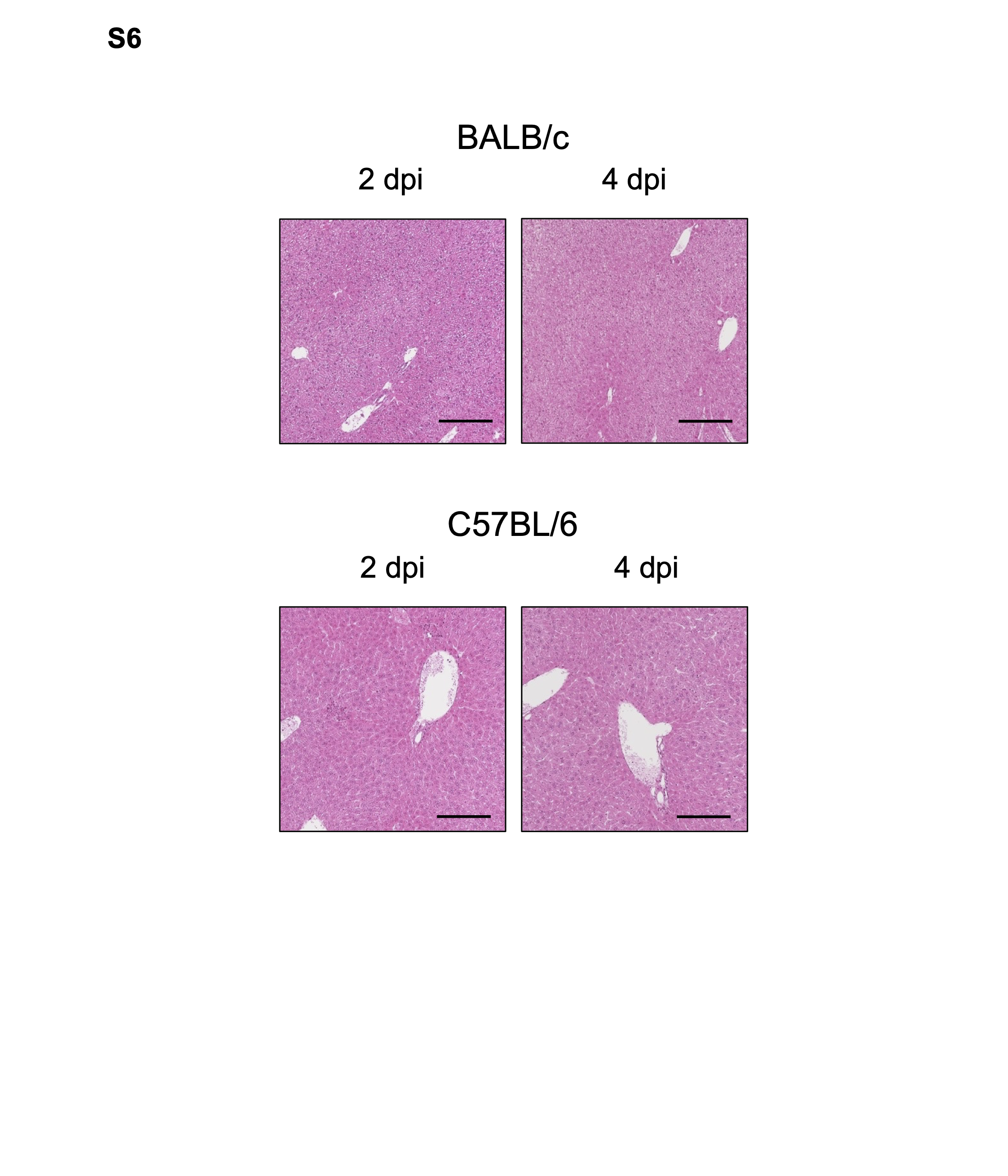

Supplement: S6 Fig — Representative H&E-stained histological images of the liver of YEZV-inoculated BALB/c mice and C57BL/6 mice at 2 and 4 dpi are shown. The scale bars on the histological images are 200 μm. (TIFF) [file ppat.1012101.s006.tiff]

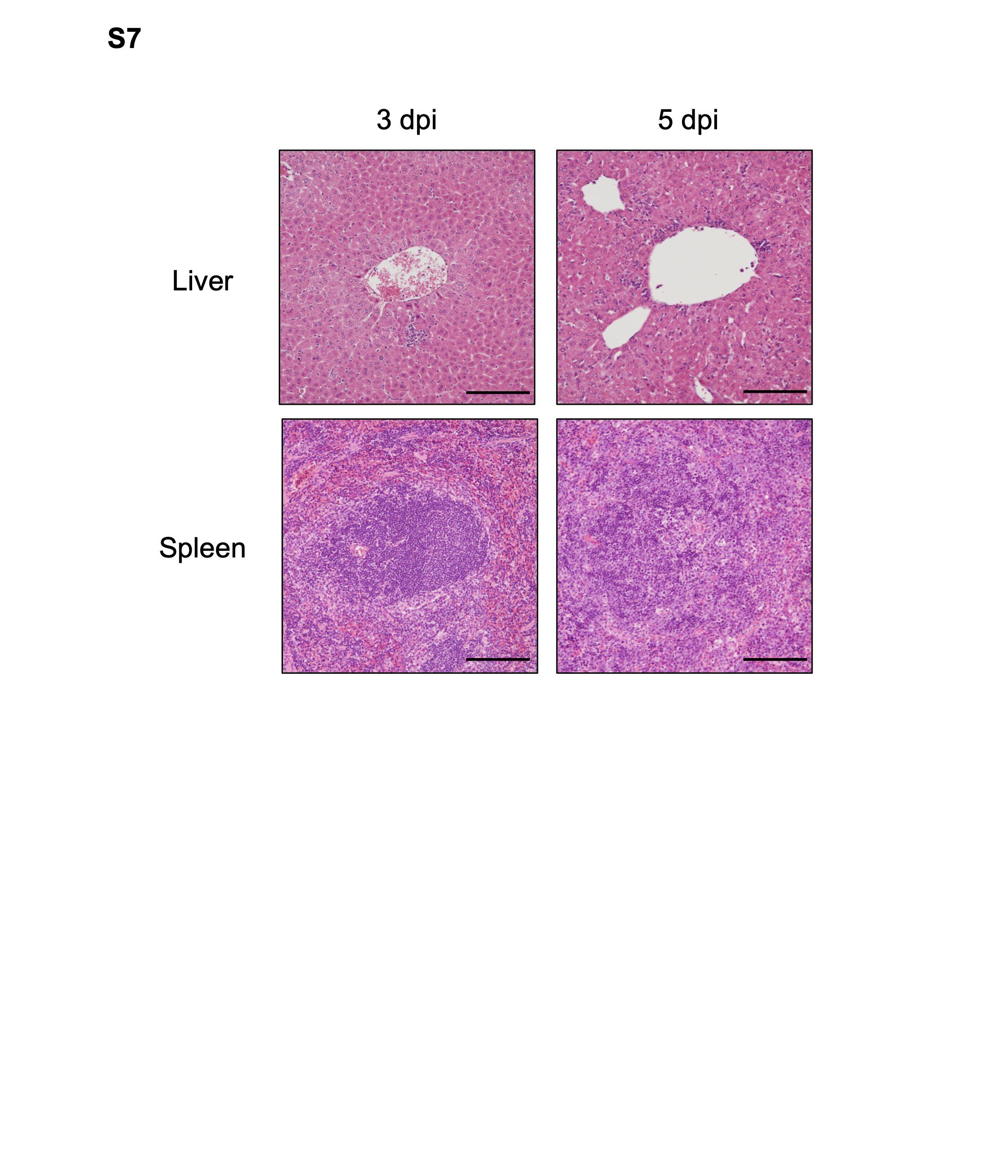

Supplement: S7 Fig — AG129 mice were subcutaneously inoculated with 104 FFU of YEZV and sacrificed for organs sampling at 3 and 5 dpi. Representative H&E-stained histological images of the liver and spleen of the mice at 3 and 5 dpi are shown. The scale bars on the histological images are 200 μm. (TIFF) [file ppat.1012101.s007.tiff]

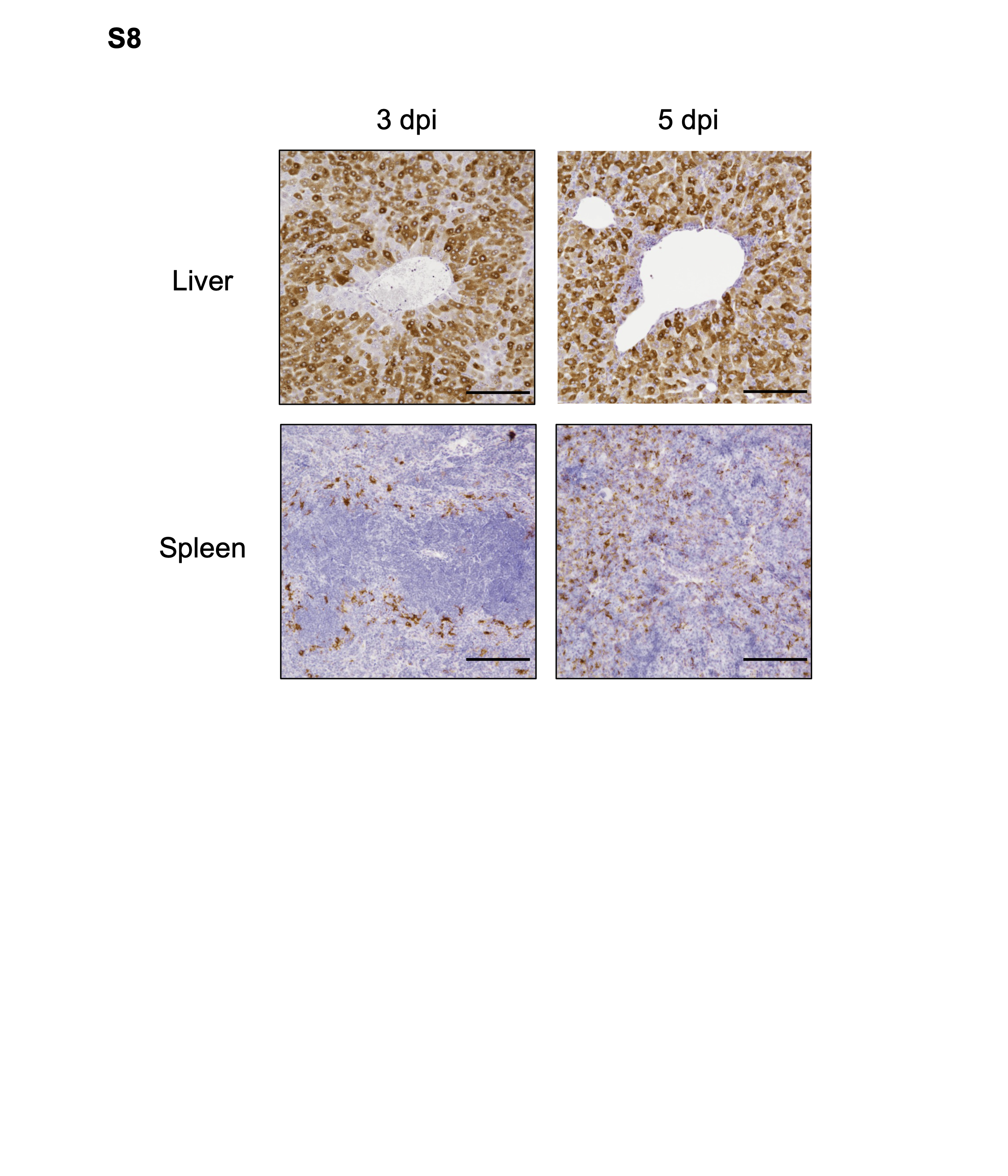

Supplement: S8 Fig — AG129 mice were subcutaneously inoculated with 104 FFU of YEZV and sacrificed for organs sampling at 3 and 5 dpi. Viral antigens were detected using anti-YEZV N protein rabbit antibody. The scale bars on the histological images are 200 μm. (TIFF) [file ppat.1012101.s008.tiff]

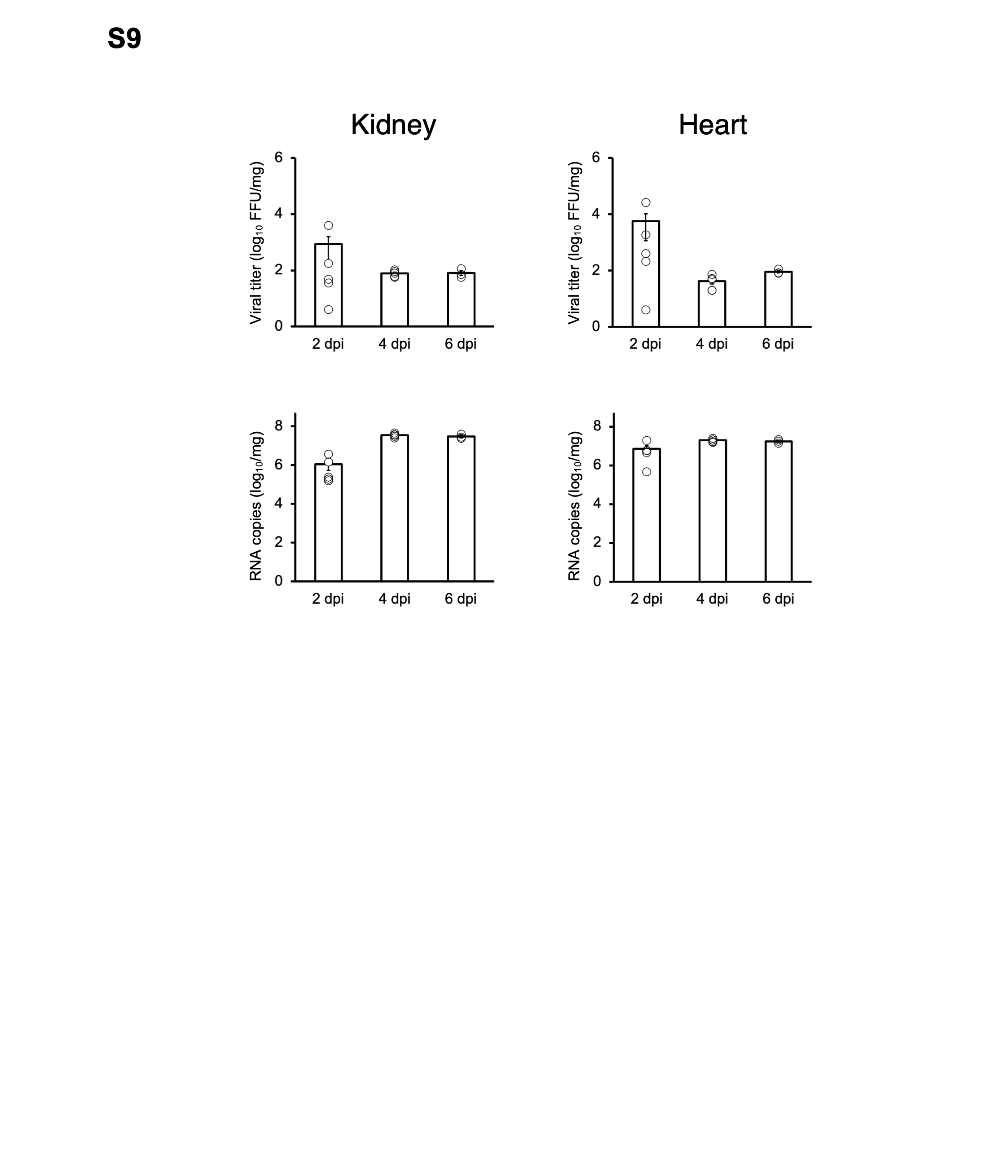

Supplement: S9 Fig — Kidneys and hearts of YEZV-inoculated AG129 mice collected at 2, 4 and 6 dpi were crushed to prepare emulsions (2 dpi: n = 5, 4 dpi: n = 5, 6 dpi: n = 3). Virus titers and viral RNA (YEZV L segment) were quantified by focus-formation assays and RT-qPCR respectively. Plots lower than the limit of detection (L.O.D.) of viral titration (4 FFU/mg) are indicated by the half value of L.O.D. on the graph. White circles, thick bars, and thin lines indicate individual values, mean values and standard errors, respectively. (TIF) [file ppat.1012101.s009.tif]

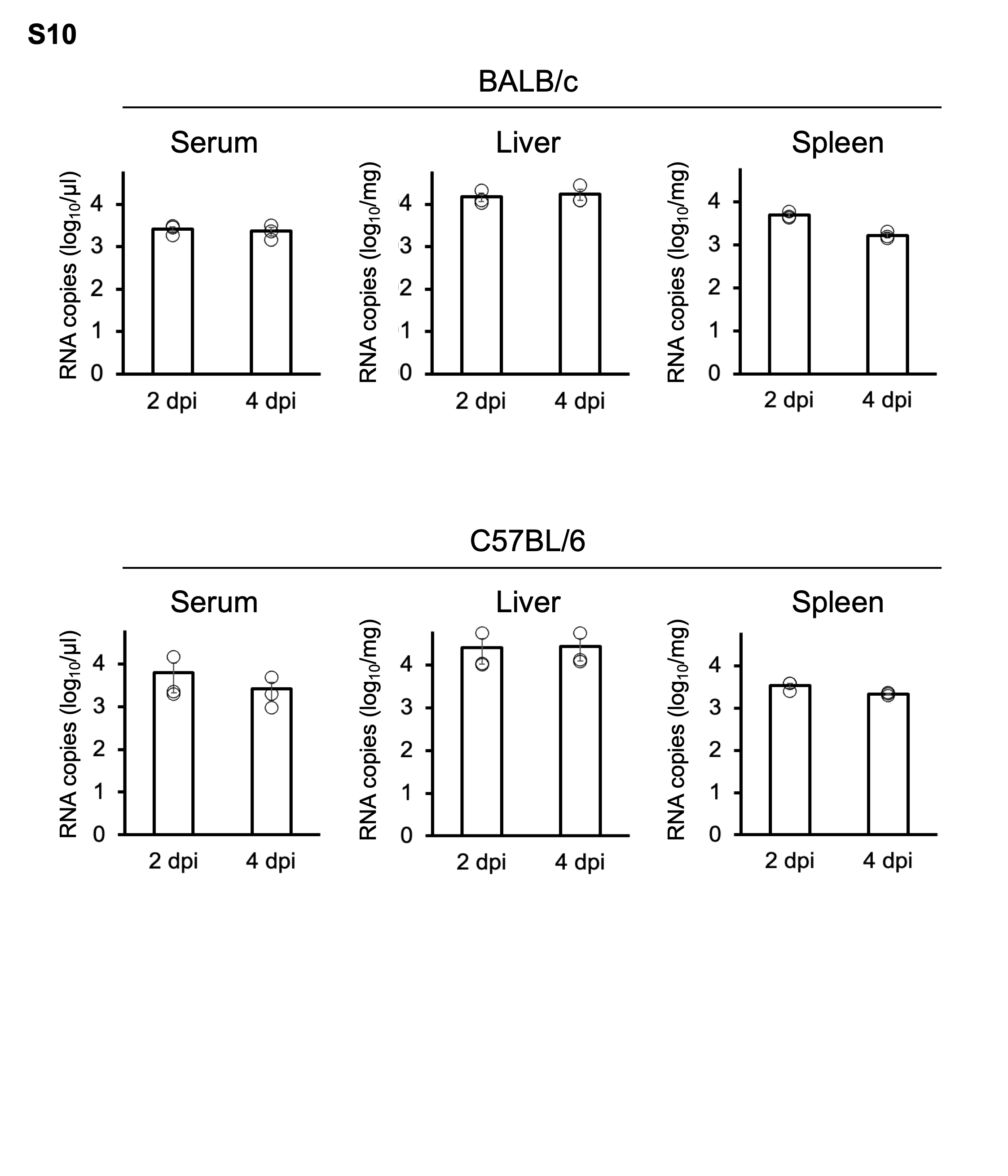

Supplement: S10 Fig — Serum, livers, and spleens of YEZV-inoculated BALB/c and C57BL/6 mice were collected at 2 and 4 dpi (2 dpi: n = 3, 4 dpi: n = 3) and subjected to RNA extraction. The amount of viral RNA (YEZV L segment) was quantified by RT-qPCR. White circles, thick bars, and thin lines indicate individual values, mean values, and standard errors, respectively. (TIF) [file ppat.1012101.s010.tif]

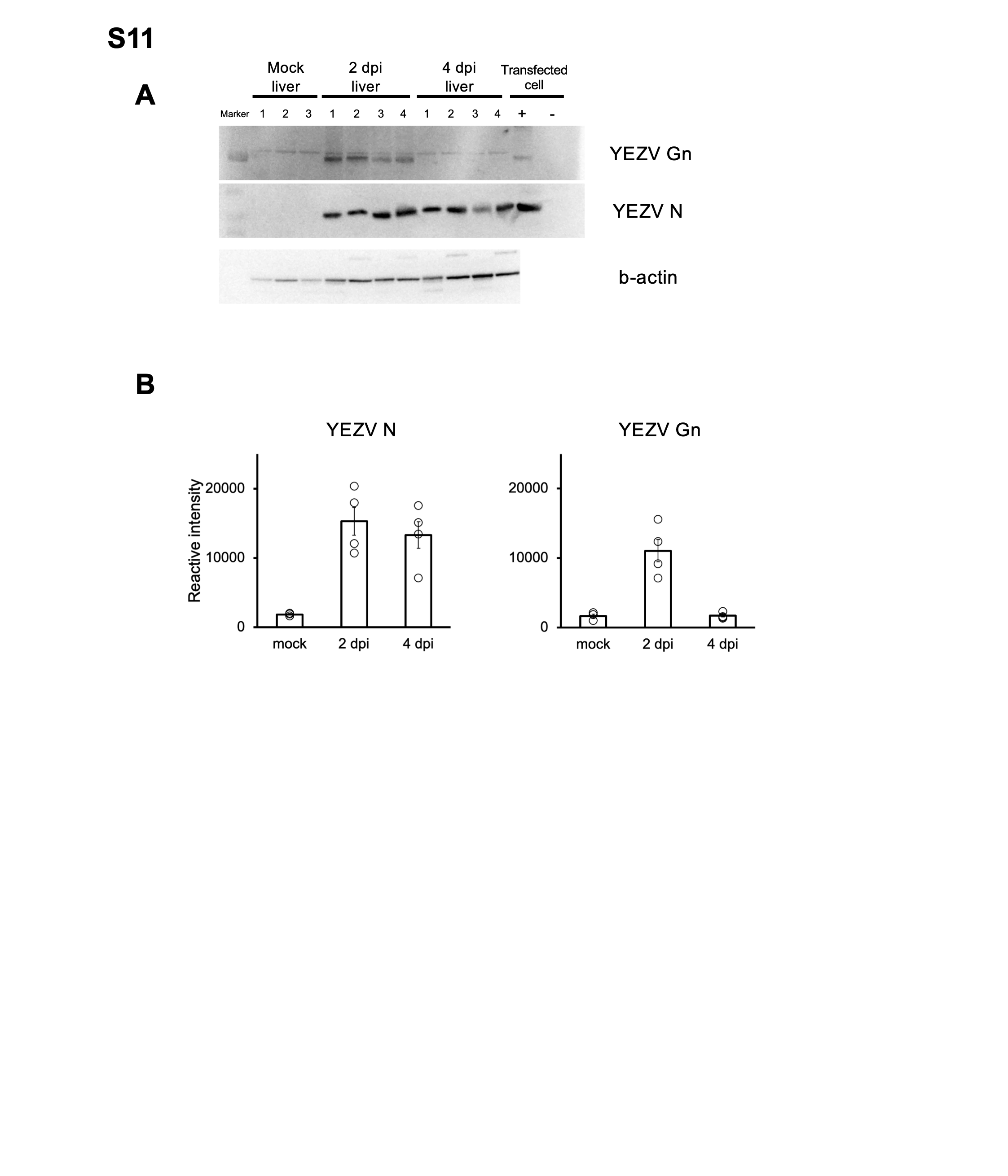

Supplement: S11 Fig — (A and B) Equal amounts (4 μg) of mock-infected and YEZV-infected mice liver emulsions were separated by SDS -PAGE. Immunoblotting analyses were conducted using anti-YEZV Gn, anti-YEZV N and anti-beta-actin antibodies. HEK 293 cells transfected with YEZV Gn or YEZV N were used as positive controls. (B) Signal intensities were quantified using ImageJ software. White circles, thick bars, and thin lines indicate individual values, mean values and standard errors, respectively. (TIF) [file ppat.1012101.s011.tif]
